# PathogenMIPer: a tool for the design of molecular inversion probes to detect multiple pathogens

**DOI:** 10.1186/1471-2105-7-500

**Published:** 2006-11-14

**Authors:** Sreedevi Thiyagarajan, Miloslav Karhanek, Michael Akhras, Ronald W Davis, Nader Pourmand

**Affiliations:** 1 Stanford University, Palo Alto, CA, USA

## Abstract

**Background:**

Here we describe PathogenMIPer, a software program for designing molecular inversion probe (MIP) oligonucleotides for use in pathogen identification and detection. The software designs unique and specific oligonucleotide probes targeting microbial or other genomes. The tool tailors all probe sequence components (including target-specific sequences, barcode sequences, universal primers and restriction sites) and combines these components into ready-to-order probes for use in a MIP assay. The system can harness the genetic variability available in an entire genome in designing specific probes for the detection of multiple co-infections in a single tube using a MIP assay.

**Results:**

PathogenMIPer can accept sequence data in FASTA file format, and other parameter inputs from the user through a graphical user interface. It can design MIPs not only for pathogens, but for any genome for use in parallel genomic analyses. The software was validated experimentally by applying it to the detection of human papilloma virus (HPV) as a model system, which is associated with various human malignancies including cervical and skin cancers. Initial tests of laboratory samples using the MIPs developed by the PathogenMIPer to recognize 24 different types of HPVs gave very promising results, detecting even a small viral load of single as well as multiple infections (*Akhras *et al, personal communication).

**Conclusion:**

PathogenMIPer is a software for designing molecular inversion probes for detection of multiple target DNAs in a sample using MIP assays. It enables broader use of MIP technology in the detection through genotyping of pathogens that are complex, difficult-to-amplify, or present in multiple subtypes in a sample.

## Background

Detection of the different types and subtypes of a pathogen is very important in clinical diagnosis. Over the past several years, the development and application of molecular diagnostic techniques has initiated a revolution in the diagnosis and monitoring of infectious diseases. Advances in molecular and cell biology have provided us with an understanding of the mechanisms of disease at the molecular level that can now be translated into designing new diagnostic, prognostic, and therapeutic tools. It is now possible to genotype/subtype pathogens to increase the accuracy and reproducibility of diagnosis. Detection of microbial DNA can provide not only a signature for the presence of a disease, but may also indicate a drug-resistant genotype, a particularly virulent subtype, a subtype associated with other clinically important sequelae, or the presence of multiple subtypes. These advances should open the way for administering more effective and efficient treatment for the control, prevention and cure of diseases. There are several multiplex detection methods currently in use, like DNA microarray, MUCH-AMASE, multiple sequencing and pyrosequencing [1-5], but they perform poorly in subtyping of pathogens in a sample, as all of them needs a pre-amplification of DNA through a PCR, and thus target a region of the whole genome where PCR primers are possible. This limits the number of target organisms or types/subtypes that can be detected in an assay. Genomes of some viral pathogens are too diverse to design a PCR primer pair whereas some pathogen genomes have types and subtypes with less diversity for designing specific probes.

Molecular Inversion Probe (MIP) technology was initially developed for the detection of single nucleotide polymorphisms (SNPs) in human genes [6,18]. MIP technology has been shown to work well for multiplexing, i.e. massive parallel processing (12,000 MIPs in the same reaction tube) [7]. The power and versatility of MIP technology makes it perfectly suited for the identification and quantification of microbes. MIP's high sensitivity and specificity in detecting large numbers of SNPs [6,7] should allow one to harness this technology to detect a large number of pathogens and to identify multiple infections in an individual sample. MIP assays are promising for a number of clinical diagnostic applications such as detection of microbial co-infections, antibiotic resistance and measurements of microbial copy number in a single tube.

**Figure 1:**
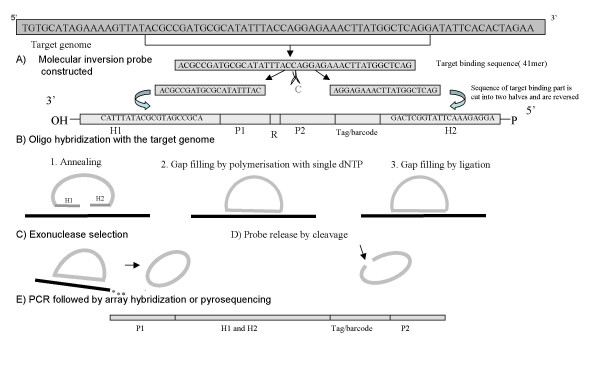
**Steps involved in a standard MIP Assay (Steps A – E)**. H1 & H2 – Two segments homologous to the target genome, P1 & P2 – Two universal primers common for all MIPs, R – Cleavage site. A) A Molecular inversion probe constructed using PathogenMIPer software, for each of the organism to be detected in the MIP assay. An oligo of user specified length is extracted from the target genome, and cut into two halves, reversed and tailored with the primer and other tags. B) Oligo hybridization with the samples. Done enzymatically – a mixture of the genomic DNA, MIP probes specific for all the targets, a thermostable polymerase and ligase, is heat denatured and brought to annealing temperature. i) Two sequences targeting each terminus of the probe hybridize to complementary sites in the genome creating a circular conformation with a single nucleotide gap between the termini of the probe. All the MIP probes are designed to have the same nucleotide at this position. ii) Unlabeled dATP/dGTP/dCTP/dTTP (any one selected by the user while designing the probe) is added to the reaction and the polymerase adds the nucleotide to the gap, and then iii) the ligase closes the gap to form a circular molecule that encircles the genomic strand, to which it's hybridized. C) Exonucleases are added to remove the excess unreacted linear probes and any linear genomic DNA. The reactions are then heated to inactivate the exonucleases. D) The probes are released from the genomic DNA by reacting with uracil-N-glycosylase. E) PCR reagents are added along with the common PCR primer pair. The reactions are subjected to thermal cycling, with the result that only circularized probes which bound to the specific target, are amplified. The probes are detected using a tag microarray or pyrosequencing.

A molecular inversion probe is comprised of genomic recognition sequences, common amplification sequences and a molecular barcode for each genotype assigned to a specific gene. This probe is a linear oligonucleotide with target-complementary sequences at the ends and a non-complementary linking segment in between (Figure 1A). Upon hybridization, the ends are brought together, creating a double helix with a gap of one or more nucleotides such that polymerization to fill the gap and subsequent ligation will occur only in the presence of a specific nucleotide. Upon appropriate filling and ligation, the oligonucleotide is circularized and, due to the helical nature of double-stranded DNA, it encircles the target DNA strand (Figure 1B). Probes that are successfully filled and ligated are amplified using universal primers (Figure 1C–E). A universal barcode sequence allows for array detection and quantification of amplified probe. This strategy provides the following advantages: i) high level accuracy is reliably obtained based on fidelity of both polymerase and ligase in the gap-fill step, ii) high specificity is ensured from hybridization, polymerization and ligation; indeed these latter enzymatic reactions together result in very specific and accurate pathogen typing. The use of a MIP in genomic detection techniques is shown in figures 1A–1E.

In order to target the entire genome of a pathogen, the probes must be very sensitive, and long enough to detect even a few copies of the pathogen DNA in the sample. The reliability of the MIP assay depends wholly on the specificity and binding efficiency of the probes. To harness MIP technology and make it available for widespread clinical use, it will be necessary for researchers to rapidly design high quality probes. The classical approach for designing oligonucleotides, that is found in existing softwares [20-24] were mainly developed for the design of microarray probes, PCR or sequencing primers. Most of these software tools use the same algorithms to design probes based on the criteria specified by the user, and these approaches are not always adapted to complex biological samples, for which multiple organism detection is needed. The available tools for MIP design [3,17] do not check for cross-hybridization with the potential background, or host genome. This is a critical factor in that cross-hybridization could lead to misinterpretation of the results in a clinical diagnosis. Here we describe PathogenMIPer, a tool for designing unique and specific probes targeting microbial (or other) genomes. In contrast to other MIP design programs, our system designs highly unique probes specific for each target sequence through multiple, successive steps of evaluation of candidates, followed by a final check against non-target genomes potentially present in the sample that could otherwise cause background noise or interfere with signal. Because of its reliability and ease of use, PathogenMIPer can be used by specialists and non-specialists alike, which would allow MIP technology to become more widely accessible.

## Implementation

A molecular inversion probe (Figure 1A) consists of two target specific binding segments, called homologue1 and homologue2, that are homologous to the target sequence and are separated by an optional single common middle base nucleotide (A, G, T, or C) in the target. In a MIP, these homologues are separated by two or more tag sequences, which include a universal primer pair for the amplification of all the target bound probes, and a unique tag which detects and identifies each of the probes after binding to the target specifically. The MIP also may contain an optional restriction or cleavage site. The total length of the MIP will be around 100 bases, depending on the lengths of target-binding homologues, primer sequence, restriction site and unique tag.

The software first generates target sequences and then in several consecutive steps eliminates unfit candidates. In order to minimize the time required to perform a design task, oligos are processed in several steps of increasing computational intensity, with a recursive elimination of unfit candidate probes at each of successive steps. The processing steps in our MIP design are as follows:

1. Candidate probes for each of the target sequences are generated with a userspecified binding length.

2. Candidates with a continuous stretch of six or more of the same base are eliminated.

3. The middle base is checked for the preferred base (A|G|T|C). Candidates without the preferred middle base are eliminated.

4. The remaining candidates are checked against non-target genomes. If an absolute match is found, the candidate is eliminated.

5. The middle 11-base region is checked against other genomes to ensure uniqueness of probes and specificity of the assay.

Automatically, the nonspecific candidates previously identified for other targets are also removed.

6. Candidates are checked for their melting temperature (Tm) and those that are beyond the desired range are eliminated.

7. Candidate probes whose similarity to non-target sequences exceeds a userspecified maximum are removed.

8. The remaining probes are screened against the potential background caused by the host genome using an online BLAST search [8] of the probes against the genome of the host.

9. In the final step, the tags are added to complete the design of ready-to-order probes. The tags are checked against the binding segments of all probes, to make sure that the addition of tags does not create a new recognizable sequence that may produce noise, false positives, or background during target detection.

Initially (step 1), all sequences of the target with the specified binding length are considered as candidate probes. Each target-specific sequence is first examined for the presence of poly As, Cs, Gs or Ts and candidates with more than 6 repeats are eliminated (step 2). After completing the check of the middle base (step 3) and an initial search to eliminate homology with non-targets (step 4), the middle 11 base region of the probe, is checked against a non-target sequence (step 5) and those with a continuous stretch of similarity with the non-target sequences are eliminated (step 6). In step 7, the candidates are checked for their Tm using the nearest neighbor method [9-11]. Surviving candidates for each target are stored in a file for later sorting and validation.

Each target sequence will have a pool of anywhere from zero to a several thousands of probe candidates. The best of the unique candidates are selected from each pool by a selective filter tool (step 8). In step 8, each of the candidates is scored for number of matching bases with the non-target sequences using a BLAST search [8]. The scoring is done in such a way that higher scores are given for matches towards the middle region of the probe, where the probe initiates binding with the target. When a candidate has a number of matching bases greater than the cutoff value, that candidate is eliminated.

The design strategy used in our software evaluates specificity at each step. Many existing oligo design tools [12-16] are difficult to customize to attain the specificity required for a molecular inversion probe. The application that most closely addresses the needs of MIP design (ProbeMaker) [11] falls short when the goal is to design unique MIPs for distinguishing between 105 HPV types at the same time. In general, there are many tools that are useful for designing MIPs for the detection of SNPs [11], but they fail when challenged to design MIPs for many subtypes of a complex genome, such as MIPs to detect 105 HPV types concurrently. Even if the number of tests or restrictions on the probe candidates is reduced, these programs tend to return a number of probes that is equal to the number of all HPV types without any uniquely selective probes (in the sense having more than 90% match in a non-target HPV type). In addition to these problems, most of these probes have close matches in the host genome, a property that is critical to remove from a probe that will be used for the detection of multiple subtypes of pathogens.

In order to design a sensitive, reliable and consistent MIP assay, all probes for each target genome in the assay must be very selective and the selectivity determinants should be located mainly in the middle of the target binding region. These criteria are not enforced in any of the existing tools. The problem is made more complicated in a real biological situation, as some parts of the genome and of the target(s) may be highly mutated, so that even probes that are designed for a consensus target with high specificity may fail to bind to the actual targets, because of natural variation. Thus a pathogen MIP design strategy should include a user option to avoid particularly variable regions of the genome during the design of target-specific probes. The PathogenMIPer software allows for such options.

**Figure 2:**
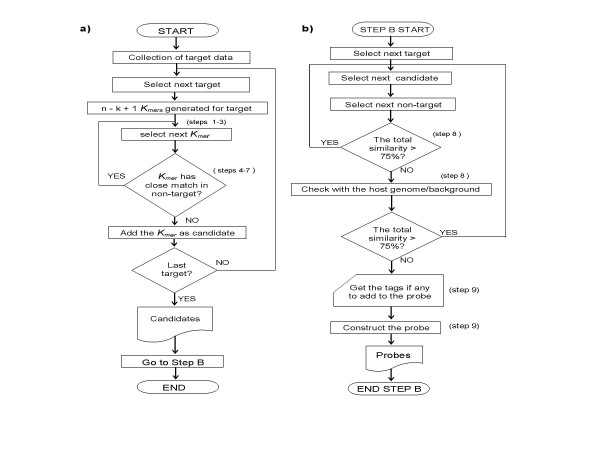
**Steps in the design of MIPs**: a) The software generates candidates for each target, based on the user specified criteria. b) The candidates are checked for their nonspecific binding with non targets and the host genome and nonspecific ones are eliminated and the specific ones are selected for designing MIP probes. n = genome length; k = user specified target binding length of probe; K*mer *= genome fragment or oligo of length = k. (Numerical value of K = k).

PathogenMIPer designs candidate probes for each target sequence through several computational steps as is shown in Figure 2. The PathogenMIPer software accepts sequence data, criteria for probe design, tags and primer sequences through a graphical user interface. After the data are validated and carefully checked, a summary view of the design is displayed and confirmed by the user to actually start a design process. The criteria that are put in by the user and accepted by the program are length of homologous sites, middle base identity, and Tm. A length in the range 40–60 is ideal for the length of homologous parts of the probe. This will give an ideal Tm of 50–60°C. The actual program runs in four stages on a PC and the results are written as a document file.

### Laboratory validation of software results

We used the PathogenMIPer software to design MIPs for 105 different types of human papilloma viruses (HPV). These genomes differ only slightly at most of the regions. Input constraints included a Tm of 60°C, length of 50 bp and middle base G. Multiple probes were designed for each target pathogen, and these probes complement different regions of the genome in order to exploit the variability of the whole genome and detect even small copies of the pathogen in the sample. In a preliminary test, twenty-four of the resulting probes (see Table 1) were used in a MIP assay designed for very sensitive detection of multiple pathogens in a sample. These 24 probes were selected in such a way, representing the 24 most common HPV genotypes, associated with human infections ranging from genital and oral warts to cervical, and oral carcinomas, as reported in several clinical studies. The clinical samples, containing extracted total genomic DNA from cervical tumor scrapes, were obtained from Oncomatrix [25].

**Table 1:** The HPV plasmid types tested in the laboratory with MIPs designed with the PathogenMIPer software.

Types of HPV plasmids tested	Accession number
HPV 6	X00203
HPV 11	M14119
HPV 16	K02718
HPV 18	X05015
HPV 31	J04353
HPV 33	M12732
HPV 34	X74476
HPV 35	M74117
HPV 39	M38185
HPV 40	X74478
HPV 42	M73236
HPV 43	NC_005349
HPV 44	U31788
HPV 45	X74479
HPV 51	M62877
HPV 52	X74481
HPV 56	X74483
HPV 58	D90400
HPV 59	X77858
HPV 66	U31794
HPV 68	NC_004710
HPV 69	M73258
HPV 73	U21941
HPV 82	X94165

Using these samples as well as purified HPV plasmids and probes designed by PathogenMIPer, we performed an initial validation in the lab to evaluate specificity and sensitivity using both MIP assay [6] and pyrosequencing [2] results as a comparator.

The samples were subjected to PCR using GP5/GP6+ primers, followed by pyrosequencing [2]. The methods for conducting the MIP assays are as described previously [6] with the minor modifications. In brief, for the initial annealing reaction, 400 ng genomic DNA or ~1–100 pg HPV plasmids, ~5 fmol of each of the 24 probes, 0.05 units Ampligase (Epicentre, Madison, WI), and 0.5 units Stoffel fragment DNA polymerase (Applied Biosystems, Foster City, CA) in 10 μL of 20 mmol/L Tris-HCl (pH 8.3), 25 mmol/L KCl, 10 mmol/L MgCl_2_, 0.5 mmol/L NAD, 0.01% Triton X-100 and 0.5 mM dGTP were incubated for 4 minutes at 20°C, 5 minutes at 95°C and then 15 minutes at 60°C, the reaction was cycled 10 cycles. Subsequently, 10 units exonuclease I and 200 units exonuclease III (Epicentre, Madison, WI) were added and the mixture was incubated for 60 minutes at 37°C and 20 minutes at 80°. Following exonucleolysis, the reaction was subjected to uracil depurination and cleavage with 2 units uracil-*N*-glycosylase (New England Biolabs, Ipswich, MA) in 20 μL of 1.6 mmol/L MgCl_2_, 10 mmol/L Tris-HCl (pH 8.3), and 50 mmol/L KCl and incubated for 60 minutes at 37°C and 20 minutes at 80°C. To amplify the inverted probes, 1.5 units AmpliTaq Gold (Applied Biosystems), 10 pmol of each universal primer in 25 μL of 1.6 mmol/L MgCl_2_, 10 mmol/L Tris-HCl (pH 8.3), 50 mmol/L KCl, and 112 μmol/L deoxynucleotide triphosphate (dNTP) were added to the genotyping reactions.

The reactions were amplified in 35 cycles of 95°C for 30 seconds, 55°C for 30 seconds, and 72°C for 10 seconds.

### Microarray preparation

Microarrays were prepared using CodeLink activated slides (Amersham, Piscataway, NJ) with 5'amine-modified oligonucleotide. Oligonucleotides were printed onto microarray slides from a solution containing 1× printing buffer (50 mM sodium phosphate, pH 8.5), 20 μM probe using a microarrayer (BioDot, Inc., Irvine, CA). Positive and negative controls were printed on the microarray as well.

Each sample was printed in 4 replicates and 2 arrays were present on each chip. The post-printing processing of the microarray-chips were performed as recommended by the slide manufacturer.

### Microarray hybridization

For the target hybridization step we used 50 μL of biotinylated PCR products, 1× hybridization buffer (100 mM MES, 1 M [Na+], 20 mM EDTA, 0.01 Tween 20) and 1.25× Denhardt's solution. The hybridization was performed at 42°C for 12–16 h. After hybridization, the microarray was washed with wash buffer (6× SSPE, 0.1% Tween 20) 2 times at 50°C for 2 min and once at room temperature for 2 min. The microarray was labeled for 10 min at 50°C with a solution containing streptavidin-allophycocyanin conjugated (1 mg/ml), 6× SSPE, 1× Denhardt's solution and 0.01% Tween 20. Following washing, 3 times in wash buffer, the microarray was assayed for fluorescent intensity at 635 nm using a GenePix 4000 fluorescent scanner (Axon Instrument, Foster City, CA) set to scan at 450 PMT. We used GenePix Pro software to determine the total fluorescent signal from each feature. To further confirm the results, 10 μl of the same PCR product that was used in microarray hybridization was sequenced by pyrosequencing as described by Gharizadeh et al [2].

The sensitivity (Sn) and specificity (Sp) of any system can be defined in terms of four outcomes – true positive (TP), true negatives (TN), false positives (FP), false negatives (FN) [17,18]. In our assay, the sensitivity is the fraction of actual positives which are predicted as positives, and specificity is the overall fraction of prediction that is correct. This correctness was checked by pyrosequencing method [2].

## Results

### Unique sequence generation

As described above, candidate homologous sequences were checked and evaluated, and the bad ones were eliminated (steps 1–6). Then, the average melting temperature of the candidates was calculated to check whether it was feasible to design MIPs with the specified Tm. Next, all candidates were checked for the middle base, and then for their Tm. The software designed total 4244 candidates for 105 HPV types, for a user specified Tm of 60°C (the software used 60 ± 4°C), length 50 bp and middle base G. On an average 40 candidates were generated for each of the 105 HPV type genomes. The number of candidates generated for each genome varied from 30 to 75 depending on their genomic variability.

### Candidate selection

As described above, in steps 4–6, the candidates for each HPV genotype target were checked against non-target genomes for their similarity. We checked the candidates of each HPV type against the genomes of other HPV types for similarity. The two homologous sites of the probes were evaluated in such a way that if they had any sequence matches in a non-target genome in the middle region, they incur much higher penalties than for the matches distal to the middle region. For each of the comparisons of the homologous sites of the probe against a non-target-genome, the total penalty score is checked against a threshold cut off value, set by the software based on the minimum mismatch percent specified by the user while setting up parameters for the design of MIPs. If the score exceeded the cutoff, the candidate was eliminated. When this filter was used against the candidate HPV probes generated in the previous step, PathogenMIPer returned a total of 2245 candidates, or 52% of the original candidates. The remaining 48% of the candidates were discarded in this step due to potential non-specific binding with non-target-genomes. The number of candidates returned per genome varied from 3 to 41 at the end of filtering.

### Screen against the host genome

Finally (Steps 7–9), the best candidates were checked against the potential background or host genome through a remote BLAST and genome database on the NCBI server. Non-specific binding was reduced by making sure that the target-specific probe would not have more than 80% matches to the non-target genomes, or in their potential host genome. For the probes developed against 105 HPV types, the filtered candidates were subjected to BLAST against the human genome (the potential host), and PathogenMIPer eliminated 61 candidate sequences based on the matches in host genome, rendering more specificity for the assay. The software returned 2184 candidate probes for all the 105 types of HPV.

### Tag placement

Once the homologous site construction is completed, the user can add predefined tag sequences to the homologous constructs. These tag sequences can be universal primers for the amplification of the binding signal, and/or molecular barcodes for the detection of the targets using tagArray (Affymetrix, Santa Clara, CA) or by sequencing methods such as pyrosequencing [2].

The tag sequences input by the user are added in the preferred order as shown in the figure 1. Figure 1 shows a well-constructed standard MIP consisting of six parts: two homologous constructs, two universal primers (one forward and one reverse), a restriction enzyme binding site and a molecular bar code sequence. These parts have to be constructed in a specific order in order for the MIP to work perfectly. This is done by the PathogenMIPer software interactively. The tags when added to the probe sequence may generate sequences complementary to probes of other targets included in the assay, resulting in cross hybridizations and oligo-dimer formations, which will render the results to be false positive. The PathogenMIPer software checks all the constructed probes, and eliminates the ones having problems in tags. The final selected probes are written in a file, saving the eliminated ones in a separate file. The user can look at the eliminated ones and can change the tags in those eliminated ones and later choose to include them in the assay. The user can follow and add individual steps or finish the design process in a single step.

### Laboratory validation

The MIP assay was performed in 7 steps as depicted in figure1(A–E). The PathogenMIPer probes detected all 24 HPV types (100% sensitivity) when tested on plasmids with no false positives (100% specificity). In more complex clinical samples, the PathogenMIPer probes were able to produce distinct signals for the detection of single as well as multiple infections. Also, all 24 HPV types were detected in clinical samples (100% sensitivity as compared to conventional genotyping results) with no false positives (as compared to conventional genotyping techniques such as pyrosequencing). The data are shown in Table 2, 3. The pyrogram data for each of the samples were on par with the results of MIP assay.

**Table 2:** The results of clinical samples tested with the HPV MIPs designed with PathogenMIPer.

Sample #	Results from MIP assay	Results from conventional genotyping
1) OM-1078	HPV-16	HPV-16
2) OM-1272	HPV-16	HPV-16
3) OM-1299	Negative	Negative
4) OM-1301	HPV-16	HPV-16
5) OM-1452	HPV-18	HPV-18
6) OM-1464	HPV-16	HPV-16
7) OM-1530	HPV-16	HPV-16
8) OM-1569	HPV-59	HPV-59
9) OM-1668	HPV-59	HPV-59
10) OM-1741	HPV-18	HPV-18
11) OM-1751	HPV-16	HPV-16
12) OM-1848	HPV-45	HPV-45
13) OM-1854	HPV-18	HPV-18
14) OM-1967	HPV-16	HPV-16
15) OM-1980	HPV-18	HPV-18
16) OM-2006	HPV-16	HPV-16
17) OM-2059	HPV-16	HPV-16
18) OM-2215	HPV-18	HPV-18
19) OM-2257	HPV-16	HPV-16
20) OM-2258	HPV-45	HPV-45

**Table 3:** Summary of results.

TP	TN	FP	FN	Sn	Sp
24	0	0	0	24/24 (100%)	20/20 (100%)

The sensitivity (Sn) and specificity (Sp) of any system can be defined in terms of four outcomes – true positive (TP), true negatives (TN), false positives (FP), false negatives (FN) [17, 18]. In our assay, the sensitivity is the fraction of actual positives which are predicted as positives, and specificity is the overall fraction of prediction that is correct. When the MIPs designed using PathogenMIPer were used in detection, it gave a sensitivity of 100% and a specificity of 100%.

### Performance

Software performance of probe design is mainly limited by the amount of available memory, and the processor speed. The initial phase of generating candidate homologous sites (Steps 1–4) takes considerable processing time. In the example described here, this stage took ~3 hours. If more constraints are applied, the processing time will be increased, as more and more testing processes are done on the probe candidates. The number of targets and the length of each target affects the processing time as well. The total processing time is also depending on the number of candidates returned in the first step. When the number of targets increases, the processing time increases exponentially. Another major factor for the processing time is the divergence of the pathogen genomes. When the genomes of pathogens included in a MIP assay are more divergent, or distant, a greater number of candidates are generated and the processing time is longer.

The second phase of evaluating the candidates (Steps 5–7), is a very computationally intensive process, and the time taken for this step (~50 minutes in our case) also depends on the parameters specified by user. The last step, in which the sequences are checked against the host genome through a BLAST, was done on the genome database at NCBI server. The criteria for BLAST are set by the PathogenMIPer software and the BLAST software called upon is the current version running remotely on the NCBI server.  Although this takes additional processing time (~9.5 hours in this example), it adds to the quality of the MIP assay by increasing uniqueness and reducing noise.

When used for designing 105 different types of human papilloma viruses, PathogenMIPer generated a complete pool of probe sequences in less than 15 hours. The software is quite flexible in allowing candidates generated in each of the steps to be viewed, checked and accessed for editing so that one can go back to any step in the process and revise the design without deleting the candidates of the previous design, although such interventions would of course be more time-consuming.

## Discussion

In preliminary tests with just 24 probes against 24 HPV genotypes, (i.e. one probe for each genotype present), we were able to sensitively detect all 24, with a lower limit of detection for purified plasmid as low as ~1 pg. In addition, we were able to detect multiple infections in samples containing HPV plasmids, without false positives, resulting in a specificity of 100%. Note that although PathogenMIPer designed 3 – 20 probes for each HPV type, this initial validation used only one probe per genotype. By relying on a single probe for the entire genome of HPV, which is ~8000 bp long, poor binding or even non-binding of the probe to the target would result, if mutations occur in the same locus chosen for the design of the MIP for these genotypes, leading to suboptimal results. A more complete diagnostic would necessarily anticipate such a problem by incorporating multiple probes for those subtypes whose genetic variation makes their identity more difficult to distinguish.

The PathogenMIPer system is extendable, with the addition of new modules and new design strategies. Because the quality requirements and other criteria for probes vary for different MIP assays, this software is able to support the design of different types of oligonucleotides, based on the constraints specified by the user. Although the processing time initially increases exponentially with the number of target genomes, this processing time levels off after a certain number of targets/genomes, as the software designs candidates by progressive elimination of non-specific candidates.

Although the PathogenMIPer software is a tool for the design of highly specific oligonucleotides for use in MIP assays, this system essentially serves as an *in silico multiplex test system*, whereby one can check the feasibility of designing a MIP assay for the specific targets one might want to include in a multiple detection technique. In the process of identifying unique specific sequences and adding tag sequences, universal primers, or cleavage sites to the complete probe design for a MIP assay, PathogenMIPer performs an efficient search for probes that have the highest probable success of producing a viable and robust MIP assay.

## Conclusions

PathogenMIPer is a software for designing molecular inversion probes for detection of multiple target DNAs in a sample. This software simplifies development of MIP assays, enabling broader use of MIP technology in the detection through genotyping of pathogens that are complex, difficult-to-amplify, or present in multiple subtypes. PathogenMIPer facilitates large scale multiplex assays, without the need for PCR sample preparation before analysis. For multiple detection strategies where amplification by a common primer is not possible, the feasibility of developing a MIP assay can be interrogated through our *in silico *system prior to launching costly and time-consuming laboratory experiments. The system can be extended to design probes for new types of assays such as hairpin inversion probes. Extending the software by adding various modules for designing different types of primers and probes is under way.

## Availability and requirements

PathogenMIPer software is available as an additional file with the article.

(See Additional files 1, 2, 3, 4, and 5).

Name: PathogenMIPer

OS: Windows

Language: Perl

Requirements: Internet connection for background checking.

## Authors' contributions

ST designed and developed the software tool and drafted the manuscript. MK checked the software and helped to draft the manuscript. MA did the wet lab for validating the probes designed with PathogenMIPer software. RD helped to draft the manuscript. NP conceived of the study, and participated in its design and coordination and helped to draft the manuscript. All authors read and approved the final manuscript.

## Supplementary Material

Additional File 1 This lists all the instructions for downloading and running the software. Click here for file

Additional File 2 This contains all the data files used for designing probes with the software and a subdirectory containing the result files obtained. Click here for file

Additional File 3 It has two software executables, needed for running the software. Click here for file

Additional File 4 It has two software executables, needed for running the software. Click here for file

Additional File 5 It has two files, needed for running the software. Click here for file
