# Supplementary material for: PathogenMIPer: a tool for the design of molecular inversion probes to detect multiple pathogens
Source: BMC Bioinformatics. 2006 Nov 14;7:500. doi: 10.1186/1471-2105-7-500 (PMC1657037; doi:10.1186/1471-2105-7-500)
Supplement: Additional File 1 — This lists all the instructions for downloading and running the software. [file 1471-2105-7-500-S1.doc]

**Download instructions:**

1. Download all three zipped files.
2. Unzip them all into the same directory, so that all the exe files are under the same directory.

Note: A data file (a sample fasta file) and a file containing tags are included for testing.

**Steps to run the PathogenMIPer application**

1. Activate/ start the software by starting the PathogenMIPer.exe .
2. Then click on “Create a new project button”. This will open an entry screen for initial parameters of MIPs design.
3. Give a new project name, folder where to create the project directory, and the project parameters – length of target binding part of the MIP, middle base, the region to be avoided while designing Mip (highly mutated/deleted part of genome)(starting and ending), melting temperature desired, fasta file where all genomes are stored.

Example of initial entry screen is on Figure 1.


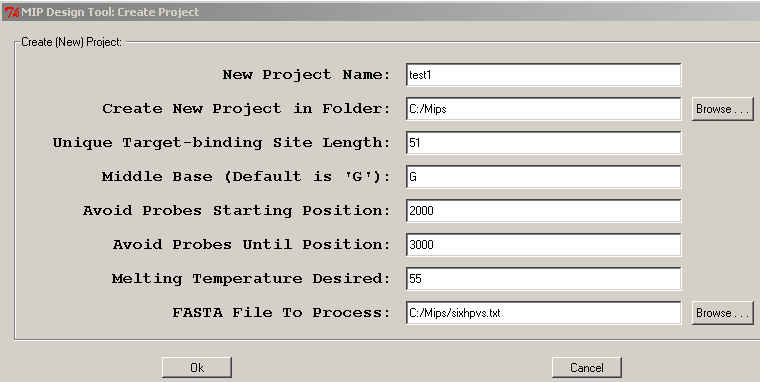


**Figure 1.** Example of creating project with initial parameters for designing MIPs candidates based on FASTA file sixhpvs.txt with 6 HPV genomes.

1. Once all parameters are set, then start the step of finding candidates for the MIPs, by pressing “Find probe candidates button”. Example of running project “test1” is on Figure 2.

**Figure 2.** Running project with initial parameters to find and generate MIPs candidates.


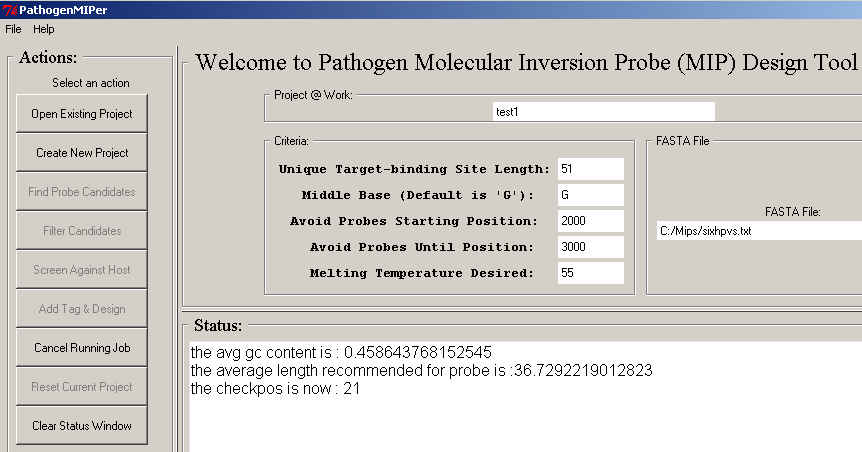


1.
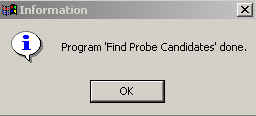
It can run for several minutes or hours depending on the genome size and number of genomes and other design parameters. Status details are shown in the status window (see Figure 2). When finding job is finished, the status is displayed through a pop-up window (Figure 3).

**Figure 3.** Popup information after finishing finding of candidates.

1. The next step is to filter generated candidates by the now enabled button “Filter Candidates”. The candidate file is located in the project subdirectory with the project name and extension “.cnd”. In our example the file name is “test1.cnd” and file is located in C:\MIPs\test1\test1.cnd. This step is giving the minimum mismatch desired for the MIPs of one target genome, against the other targets in the assay (20 – 30% is ideal. The maximum that can be set is 50%).

Example of this entry window is on Figure 4.


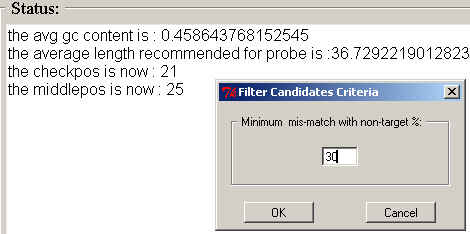


**Figure 4.** Popup entry window for filtering candidates with minimum percentage mis-match set to “30%” within target genomes.

1. When filtering candidates is finished a pop-up will inform user about its completion, see Figure 5. Filtered candidates are formatted in new file of the project directory. In our example the file name is test1.ftr located in C:\MIPs\test1\test1.ftr.


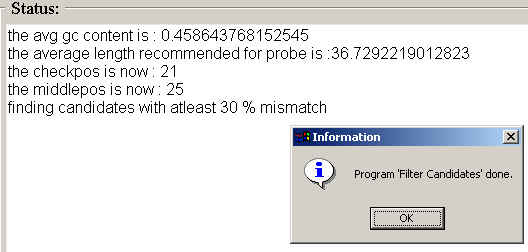


**Figure 5.** Popup information after finished filtering of candidates with 30% minimum mis-match.

1. Next step is blasting all the MIPs against the genome of potential host, or the background genome. Click the “Screen against host “ button, and give the name of host, e.g. scientific name or biological name: Homo sapiens or human, see Figure 6.


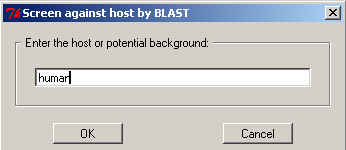


**Figure 6.** Popup window for screening candidates against “human” genome.

1. Blasting will run for a long time, because it is done remotely on the NCBI server against the database chosen by the user, see example on Figure 7.. Completion of the program is informed by the popup, see Figure 8 .


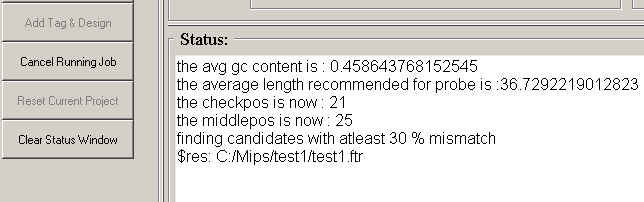


**Figure 7.** Example of running remote blast of test1 project with filtered FASTA candidates located in file C:\Mips\test1\test1.ftr.

**Figure 8.** Popup information after finishing blasting of candidates against “human” genome.


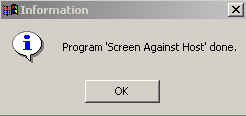


1. In the next step tags and primers are added to final probes as well as a cleavage site. This can be done by pressing “Add tag and design” button. In the input window (Figure 9) give the name of the tagfile, the universal primers in 5' -3' direction (very important), and the cleavage sequence in 5'-3'. The tagfile should be in the format, tagID followed by tag sequence separated by colon (:). The tag file should have one tag/barcode for each of the genomes included in the fasta file for the assay.

**Figure 9.** Input parameters for adding final tags to MIP probes.


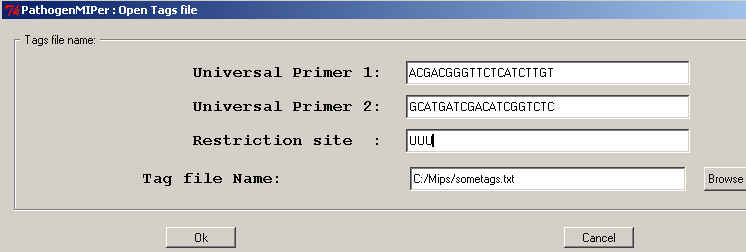


1. After adding tags is completed, program informs user by the pop-up and shows names of generated probes in the status window (Figure 10). Final file test1.prb is located in project subdirectory (e.g. C:\Mips\test1\test1.prb) as usual.


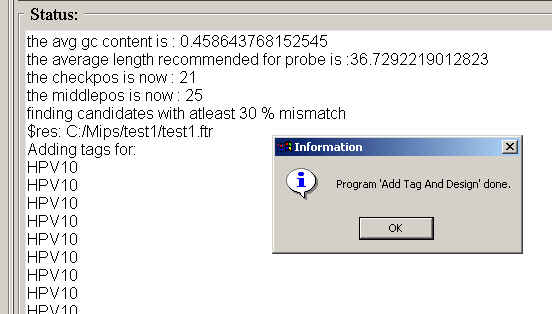


**Figure 10.** Information popup after finishing adding tags and primers to probes.

The whole design processing does not need to be done in a single stretch. Program can be stopped after finishing of any step. You can always open a project and continue working on the project until finishing all steps.

You can view the intermediary results of the progress during the design process. There is an intermediary file created after each step during the design. The file created at the end of “find candidates” has name: “project_name.cnd”. The file at the end of “filter candidates” has name: “project name.ftr”. The file after “screen against host” has name “project_name.res”, and finally the file after “adding tags” has name “project_name.prb”. After adding tags, the probes with problems in the tag region (primers or molecular barcode) are put in a separate file with name, “project_name_bad.prb”. None of these files are supposed to be edited by the users, because this will halt the further steps in the design process.

The data in the final resulting file, i.e. the probe file are tab separated and are easily viewed as a word document. They can be imported into an excel file for further use by the user. Each of the intermediary files have the genotype accession number or the id number with some features of the sequence and the probe sequence itself. The finally assembled ready to order probes rendered in the .prb file have the details in the order - genotype / id, location of the MIP in target genome, tagID, homologue2 of the probe, followed by the two primer sequences separated by the cleavage/restriction sequence, and the homologue 1 of the probe.
